# Hospitals and the Law in 1910

**Published:** 1911-01-28

**Authors:** 


### SPECIAL INSTITUTIONAL ARTICLE.

### II.—HOSPITALS AND THE LAW IN 1910.

### RATES AND THE QUESTION OF LIABILITY.

The year which has just come to a close cannot be said to have produced a very prolific crop of legal cases which are of interest to hospital authorities. Fortunately for itself the hospital as a body does not often figure as a litigant in the courts of common law. The patient who is treated gratuitously in the wards is not encouraged to bring an action if the treatment is unsuccessful, and a case decided in 1909 has shown the virtual futility of any such action. In courts of equity, on the other hand, the hospital is sometimes represented as a beneficiary, or would-be beneficiary, under the will of some "pious founder" who has not been too careful in specifying the precise object of his beneficence.

#### The Weir Charity Case.

One case of this kind was reported in 1910. We refer to the Weir case, upon which we commented last summer. There is, therefore, no need to enter into the subject in detail again. It is never a grateful task to look a gift horse in the mouth, but we venture to hope that the Weir case may serve to remind testators who leave money to hospitals that a few guineas judiciously spent in having a will properly drawn may save the expenditure of a large sum in law costs.

#### The Harrogate Poor Rate Case.

A question which is of considerable importance to hospital authorities was considered at the West Riding Quarter Sessions last summer. We refer to the method by which the rateable value of a hospital is to be ascertained. The trustees of the Bath Hospital and Rawson Convalescent Home, Harrogate, successfully appealed against the poor rates levy. The local authority had fixed the assessment at £727, which the trustees appealed against on the ground that it was far too high, and that any such assessment should be merely nominal, having regard to the work which these institutions do for the community in the district in which they are placed. The institution in question was formerly rated at £50 gross. This was subsequently increased to £500, but on appeal was reduced by the Assessment Committee to £50, and was then again raised to £727. Evidence was given that the institution was not capable of making a profit, and that the assessment ought to be nominal. It appeared that the assessment had been raised in consequence of a circular from the Local Government Board calling attention to these small assessments. In the course of the proceedings it was ruled that the case should proceed in order to decide the broad question whether the assessment should be a nominal one or not. The assessment of £727 was arrived at by taking the original cost of the buildings at £36,356, from which cost the surveyor had estimated the present value at £28,350; the site of $4\frac{3}{4}$ acres was valued at £1,694 per acre, with an annual value of £160, plus 2 per cent., calculated on the value of the buildings, or £567. This gave a total gross annual value of £727 and a net value of £582. The surveyor for the assessment authorities when cross-examined as to the beneficial occupation admitted no profit was made, and did not prove any beneficial occupation. After hearing evidence and counsel the magistrates decided unanimously to reduce the rate to the nominal sum of £50 gross and £40 net.

This decision appears to us to accord with justice and good sense, particularly when one bears in mind the principle which underlies the law of rating, namely, that the ratepayer is to be taxed according to what a hypothetical tenant would pay by way of rent for the premises when let from year to year. This principle presumes some kind of beneficial occupation, but who can say that a voluntary hospital is beneficially occupied? Many years ago it was laid down with reference to the Dreadnought Hospital at Greenwich that when dealing with a voluntary hospital the rating authorities should confine the assessment to those portions of a hospital which are beneficially occupied—*i.e.* to the houses or quarters of the resident officers and medical school buildings, and the wards for paying patients, if such there be. Bearing this wholesome principle in mind many hospital authorities will be able to resist the payment of exorbitant contributions to the local rates.

#### Consent to Operation in Minors.

While reviewing the legal decisions of the past year it is not unimportant to refer to cases which have occupied the attention of Canadian judges. The common law applied in Canada is founded on, and is for the most part the same as our own. In one case which was reported in the *International Hospital Record* (December 15) a question of general interest was discussed—namely, What is the liability of a hospital official who operates on a minor (*i.e.* a person under 21) without the consent of the parents? An action was brought by one Alfred Booth against the Toronto General Hospital and one of the surgeons at the hospital for damages for injury to his son. It appeared that the son, a youth of 19 years old, was under treatment at the hospital, where a polypus of the nose was removed. The patient having recovered, the house physician found he was suffering from a marked goître, and advised the removal of one lobe of the thyroid. This operation was carried out with the consent of the youth, but his parents were not consulted. They therefore brought an action.

The ground of action and the reasons for the decision are sufficiently disclosed in the following extracts from the judgment of Sir Glenholme Falconbridge: "I find as a fact that it is not true, as put forward, that this operation was performed without the consent of the plaintiff, that is, the young man, because at the time this action was brought he was still an infant under the age of twenty-one years, and his mother was a party to the action. I think it is manifest upon his own evidence and upon the view of what he stated in his examination for discovery, that his consent was given. What was being done for him was entirely for his benefit. The original operation was for a growth in the nose—I think it was described as a polypus—and what was done in the further operation, the removal of the thyroid, was in best of faith done for his benefit, performed—and now I turn to the position of the individual defendant, Mr. Cameron—performed without any fee or reward whatever, but entirely as a matter of benevolence; and, as I said before, his skill in the performance of the operation has never been called in question. Upon the case as presented by the plaintiff himself there is absolutely no case made out of any harm happening to this young man by reason of that operation. I doubt very much whether there is any real or satisfactory evidence of any impairment to his health. The only possible way in which it was suggested, even faintly, that his health has been impaired is that these attacks of epilepsy are said to recur at shorter intervals. No physician or surgeon stepped into the box and said that that was a possible result of the operation on the thyroid. So that I hold the case has been fully answered.

"The only question of law as to which I desired the assistance of counsel was whether in the case of a young man within three months of nineteen years of age, well grown, it was possible that the law was that a surgeon would not venture to operate upon him without getting the consent of his parents. No such law has been found for me, and I shall not be the first judge to declare that there is such a law. Of course, if it was the case of an infant of tender years, or the case of a person manifestly of weak intellect, it might be another matter; but this young man, while, as I said before, he was manifestly not of the brightest intelligence, yet he is a young man who is apparently well enough able to take care of himself; and I hold as far as this case is concerned that it is no part of the duty of the hospital authorities nor of the operating surgeon to make an inquiry or obtain any leave or license from the parent or guardian. The result is the defendants are entirely exonerated and vindicated, and the action must be dismissed with costs if costs are asked for."

So far as we are aware, the law as here stated is the same as the law which would be applied in this country. A surgeon would be reluctant to operate on a child without the consent of its parents; but if he did so, and the jury found that he did so with the consent of the child in the genuine attempt to save the child's life or to render it free from pain, no court would award damages. A further question is suggested by this case—namely, How far is a surgeon liable if he operates without the consent of his patients, whatever the age of the patient? It is clear, of course, that the surgeon who so acts commits an assault for which, if there was no conceivable justification, he would be liable. But circumstances may arise in which it is impossible to obtain the patient's consent. What, then, is the legal position? Strange to say there is only one case on the subject, and even that was not reported in the Law Reports, but is to be found in the Transactions of the Medico-Legal Society for last year. The facts there were that when the plaintiff was undergoing an operation to which she had consented, the defendant, a surgeon, thought it necessary to perform a further operation. As a result, the plaintiff sued him for damages. A jury having found that the plaintiff had not specially forbidden the performance of the second operation, the defendant was held entitled to judgment. It is probable, however, that if the plaintiff had been able to prove that she had specifically forbidden the defendant to perform the second operation, she could have recovered damages, even if he had been able to prove that the second operation was necessary for the purpose of saving or prolonging life.

